# Optimising chemical named entity recognition with pre-processing analytics, knowledge-rich features and heuristics

**DOI:** 10.1186/1758-2946-7-S1-S6

**Published:** 2015-01-19

**Authors:** Riza Batista-Navarro, Rafal Rak, Sophia Ananiadou

**Affiliations:** 1 National Centre for Text Mining, Manchester Institute of Biotechnology, 131 Princess St, Manchester, M1 7DN, UK; 2 Department of Computer Science, University of the Philippines Diliman, Quezon City, 1101, Philippines

**Keywords:** Chemical named entity recognition, Text mining, Sequence labelling, Conditional random fields, Feature engineering, Configurable workflows, Workflow optimisation

## Abstract

**Background:**

The development of robust methods for chemical named entity recognition, a challenging natural language processing task, was previously hindered by the lack of publicly available, large-scale, gold standard corpora. The recent public release of a large chemical entity-annotated corpus as a resource for the CHEMDNER track of the Fourth BioCreative Challenge Evaluation (BioCreative IV) workshop greatly alleviated this problem and allowed us to develop a conditional random fields-based chemical entity recogniser. In order to optimise its performance, we introduced customisations in various aspects of our solution. These include the selection of specialised pre-processing analytics, the incorporation of chemistry knowledge-rich features in the training and application of the statistical model, and the addition of post-processing rules.

**Results:**

Our evaluation shows that optimal performance is obtained when our customisations are integrated into the chemical entity recogniser. When its performance is compared with that of state-of-the-art methods, under comparable experimental settings, our solution achieves competitive advantage. We also show that our recogniser that uses a model trained on the CHEMDNER corpus is suitable for recognising names in a wide range of corpora, consistently outperforming two popular chemical NER tools.

**Conclusion:**

The contributions resulting from this work are two-fold. Firstly, we present the details of a chemical entity recognition methodology that has demonstrated performance at a competitive, if not superior, level as that of state-of-the-art methods. Secondly, the developed suite of solutions has been made publicly available as a configurable workflow in the interoperable text mining workbench Argo. This allows interested users to conveniently apply and evaluate our solutions in the context of other chemical text mining tasks.

## Background

In carrying out scientific work, most researchers rely on published information in order to keep abreast of recent developments in the field, to avoid repetition of work and to guide the direction of current studies. This is especially true in the field of chemistry where endeavours such as drug discovery and development are largely driven by information screened from the copious amounts of data available. Whilst databases storing structured chemical information have proliferated in the last few years, published scientific articles, technical reports, patent documents and other forms of unstructured data remain to be the richest source of the most current information.

Text mining facilitates the efficient distillation of information from the plethora of scientific literature. Whilst most of the scientific text mining efforts in the last decade have focussed on the identification of biomedical entities such as genes, their products and the interactions between them, the community has recently begun to appreciate the need for automatically extracting chemical information from text. Applications in chemoinformatics, drug discovery and systems biology such as automatic database curation [1], compound screening [2], detection of adverse drug reactions [3], drug repurposing [4] and metabolic pathway curation [5] are facilitated and informed by the outcomes of chemical text mining, a fundamental task of which is the recognition of chemical named entities.

Chemical named entity recognition (NER), the automatic demarcation of expressions pertaining to chemical entities within text, is considered a challenging task for a number of reasons. First, chemical names may appear in various forms, ranging from the popular and human-readable trivial and brand names to the more obscure abbreviations, molecular formulas and database identifiers, to long nomenclature-conforming expressions, e.g., International Union of Pure and Applied Chemistry (IUPAC) names and Simplified Molecular-Input Line-Entry System (SMILES) strings [6-8]. Moreover, researchers working on lead compound identification and discovery sometimes tend to report their results using their own arbitrarily assigned abbreviations, further aggravating the proliferation of chemical names. Also considered a barrier to the development of chemical named entity recognisers is the relatively small number of available supporting corpora, compared to those developed for biological, such as gene and protein, name recognition [9]. Whilst a few notable data sets containing chemical named entity annotations have been developed, there was a lack of publicly available, wide-coverage, large-scale gold standard corpora of scientific publications. Although the SciBorg corpus [10,11] contains a substantial number of manually annotated chemical names in its 42 full-text articles, it had not been publicly available until very recently. In contrast, the large-scale CALBC corpus [12] is publicly available, but is considered "silver standard" as it contains annotations resulting from the harmonisation of the outputs of five different automatic tools, rather than manual annotations. The similarly publicly available SCAI pilot corpus [13,14] contains gold standard annotations for various types of chemical names but is relatively small with only 100 MEDLINE abstracts.

This limited number of resources has influenced the means by which the state-of-the-art chemical named entity recognisers have been developed and evaluated. Built as a pipeline of several Markov model-based classifiers, the publicly available OSCAR tool [15] was tuned to recognise the annotation types defined in the SciBorg corpus. The system was evaluated by means of three-fold cross validation on this corpus as well as on a bespoke data set of 500 annotated MEDLINE abstracts. ChemSpot [16], another publicly available chemical named entity recogniser, is a hybrid between methods for dictionary matching and machine learning. For capturing brand names, this tool uses a lexicon-based approach for matching expressions against the Joint Chemical Dictionary [17]. For recognising nomenclature-based expressions, however, it employs a conditional random fields (CRF) [18] model trained on the SCAI corpus subset that contains annotations for only IUPAC names. The developers carried out a comparative evaluation of ChemSpot and OSCAR on the SCAI pilot corpus, in which the former was reported to have outperformed the latter by a margin of 10.8 percentage points. It is worth noting, however, that both of these tools have not been comparatively evaluated nor benchmarked against any large-scale, gold standard corpora.

In aiming to alleviate these issues, the Critical Assessment of Information Extraction in Biology (BioCreative) initiative organised a track in the Fourth BioCreative Challenge Evaluation workshop to encourage the text mining community to develop methods for chemical named entity recognition, and enable the benchmarking of these methods against substantial gold standard data [19]. Known as CHEMDNER, this track publicly released a large corpus of documents containing manually annotated chemical named entities. The 10,000 MEDLINE abstracts in the CHEMDNER corpus [20], which were grouped into disparate sets for training (3,500), development (3,500) and testing (3,000), came from various chemical subdomains including pharmacology, medicinal chemistry, pharmacy, toxicology and organic chemistry. Each annotated chemical name was labelled with one of the following mention types: systematic, trivial, family, abbreviation, formula, identifier, coordination and a catch-all category. The corpus served as the primary resource for the two CHEMDNER subtasks, namely, chemical entity mention recogniton (CEM) and chemical document indexing (CDI). Whilst the former required participating systems to return the locations of all chemical mention instances found within a given document, the latter expects a ranked listing of unique mentions without any location information.

Having participated in the CHEMDNER challenge, we have developed our own chemical named entity recogniser that obtained top-ranking performance in both the CDI (1st) and CEM (3rd) tasks. Extending that work, we describe in this paper the details of our proposed methods for optimising chemical NER performance. In the next section, we compare the performance of our methods with the state of the art and present results of our evaluation on several corpora. Furthermore, we share details on how our contributions, publicly available as a service, can be accessed and utilised by the community. The Experiments section contains a detailed discussion of our proposed methods and the experiments we have performed in order to identify the optimal solution on each of the data sets considered. We summarise the results of our work in the Conclusions section. Lastly, we provide some technical background on the techniques and evaluation metrics we have used in this study in the Methods section.

## Results and discussion

We developed a conditional random fields (CRF)-based method for chemical named entity recognition whose performance was optimised by (a) the selection of best-suited pre-processing components, (b) the incorporation of CRF features capturing chemistry-specific information, and (c) the application of post-processing heuristics.

We begin with describing the results from the evaluation of our method under the settings of the CHEMDNER challenge. Next, we demonstrate that our method obtains competitive performance compared to the state of the art. We then show that a statistical model trained on a large-scale, gold standard corpus such as CHEMDNER is suitable for recognising chemical names in a wider range of corpora, on which it consistently outperforms two known chemical NER tools. Finally, we describe the availability of our approach as a configurable workflow in the interoperable text mining platform Argo [21]. Hereafter, we refer to our suite of solutions collectively as Chemical Entity Recogniser, or ChER.

### Performance evaluation under the CHEMDNER challenge settings

The first set of experiments was performed based on the specifications of the BioCreative IV CHEMDNER track [19], which our research team participated in. The micro-averaged results on the CHEMDNER test set obtained by our solutions using specialised pre-processing analytics (i.e., Cafetiere Sentence Splitter and OSCAR4 Tokeniser) are presented in Table 1. These closely approximate the results which were reported for our submissions during the official BioCreative challenge evaluation [22], in which the variant employing knowledge-rich features and abbreviation recognition achieved the best performance in both the CEM and CDI subtasks.

**Table 1:** Performance of ChER under the BioCreative IV CHEMDNER track setting.

Custom Features	Post-processing		CEM			CDI		
	
	**Abbr**.	**Comp**.	P	R	F_1_	P	R	F_1_
✓	✗	✗	92.76	81.02	86.49	91.39	85.29	88.23
✓	✓	✗	92.76	81.30	86.65	91.37	85.45	88.31
✓	✗	✓	92.14	81.41	86.44	90.55	85.72	88.07
✓	✓	✓	92.14	81.69	86.60	90.53	85.88	88.14

Key: Abbr. = Abbreviation recognition, Comp. = Chemical composition-based token relabelling

### Performance comparison against state-of-the-art methods

We conducted a performance-wise comparison of our solution, ChER, against previously reported machine learning-based chemical NER methods, namely, that of Corbett et al. [15], Rocktäschel et al. [16] and Nobata et al. [23]. To facilitate a fair comparison, we performed a series of benchmarking tasks under the same experimental settings used in their previously reported work.

Following Corbett et al. [15], we performed three-fold cross validation on the SciBorg corpus, taking into consideration only annotations for mentions of chemical molecules. As summarised in Table 2, the F_1 _score obtained by our methods (79.66%) is slightly lower than that reported by Corbett et al. (81.20%). We cannot remark on precision and recall, however, as the authors did not report them. It is worth noting that their work became the foundation of what is now known as the OSCAR chemical NER tool. Although the software is freely available [24], we have not been able to replicate their reported results on the SciBorg corpus as the models bundled with the downloadable release were trained on documents from the same data set.

**Table 2:** Comparative evaluation of ChER against state-of-the-art chemical name recognition methods.

SciBorg (chemical molecules)				SCAI-100 (systematic names)			
	**P**	**R**	**F_1_**		**P**	**R**	**F_1_**

ChER	85.96	74.22	79.66	ChER	86.70	67.50	75.90
OSCAR	-	-	81.20	ChemSpot	57.47	67.70	62.17

OSCAR's F_1 _score was taken from the paper of Corbett et al. [15].

Following the experimental setup employed by Rocktäschel et al. [16] in evaluating their ChemSpot tool, we trained a CRF model on the SCAI training corpus containing annotations for systematic names. Consequently, the version of ChER driven by this particular model can recognise only systematic names, and was thus evaluated only against the gold standard systematic name annotations in the SCAI pilot corpus of 100 abstracts (SCAI-100). The results shown in Table 2 indicate that whilst ChER and the CRF-based component of ChemSpot achieve similar recall (67.50% and 67.70%, respectively), the former obtains far more superior precision and F_1 _score (86.70% and 75.90%) over the latter (57.47% and 62.17%). We note that in conducting this comparison, we ran ChemSpot [25] on the SCAI-100 corpus ourselves, enabling its capability to recognise multiple chemical name subtypes, in order to segregate recognised systematic names.

Last in this series of evaluations is the performance-wise comparison of ChER with MetaboliNER [23], a tool based on a CRF model that utilised the Chemical Entities of Biological Interest (ChEBI) [26] and Human Metabolome (HMDB) [27] databases as dictionaries. The tools were evaluated on the NaCTeM Metabolites corpus [28] in a 10-fold cross validation manner [23]. The obtained results, presented in Table 3, indicate that MetaboliNER achieves higher precision (83.02% vs. 81.42%); however, it is outperformed by our method in terms of recall and F_1 _score (79.66% and 80.53% vs. 74.42% and 78.49%).

**Table 3:** Comparative evaluation of ChER against a state-of-the-art metabolite name recognition method.

NaCTeM Metabolites			
	**P**	**R**	**F_1_**

ChER	81.42	79.66	80.53
MetaboliNER	83.02	74.42	78.49

We surmise that our solution's superior performance over the similarly CRF-based ChemSpot and MetaboliNER tools can be explained by the richer feature set we employed in developing ChER. As described in the Experiments section below, ChER utilises a comprehensive set of character and word *n*-grams as well as orthographic features, which were then augmented with ones which capture chemical knowledge, e.g., number of chemical basic segments, dictionary and chemical symbol matches. Meanwhile, ChemSpot employs only size-two affixes, a check for leading or trailing whitespace, a quite limited set of orthographic features and bag-of-words [16]. MetaboliNER uses a similar feature set, with the addition of word shape, part-of-speech tags and dictionary features [23]. Based on the evaluation presented, ChER's rich feature set proved to be more informative and powerful over that of ChemSpot and MetaboliNER.

### Performance evaluation on a variety of chemical corpora

As stipulated earlier, one of the barriers to the development of chemical named entity recognisers was the lack of publicly available, wide-coverage, large-scale gold standard corpora. The public release of the CHEMDNER corpus directly alleviates this issue, allowing us to train our CRF model on a massive number and variety of learning examples. We argue that a model trained on the CHEMDNER corpus produces satisfactory NER performance even on documents of different types (e.g., patents, DrugBank descriptions) and from various specialised subject domains (e.g., pharmacology, metabolomics). In validating this, we utilised the CHEMDNER training and development sets to train CRF models under the various configurations detailed in the Experiments section. Taking the best performing variant, we compared its performance with that of OSCAR and ChemSpot by also running their latest versions (OSCAR4.1 and ChemSpot 2.0) on each corpus of interest. Across all five corpora we used, ChER consistently outperformed the other two NER tools, often with a noticeable margin.

Presented in Table 4 are results of this evaluation scheme on general chemical corpora. On the SCAI-100 corpus, with all chemical name types taken into consideration, ChER achieved a good balance between precision and recall, giving an F_1 _score of 78.27% which is almost four percentage points higher than that of the second-best performing ChemSpot. An even larger margin of about 12 percentage points (also in terms of F_1 _score) was obtained by ChER over ChemSpot on the Patents corpus [29,30]. The relatively low F_1 _score on this corpus (64.75%) can be explained by the difference in document types between the corpus for model training (i.e., scientific abstracts) and evaluation (i.e., patent applications). We note that an evaluation on a third chemical corpus, SciBorg, was not carried out under this scheme. Since OSCAR was trained on the SciBorg corpus, a comparative evaluation of ChER, OSCAR and ChemSpot on this data would not have given fair results.

**Table 4:** Applicability of ChER with the CHEMDNER model to other chemical corpora.

	SCAI-100 (all names)			Patents		
	**P**	**R**	**F_1_**	**P**	**R**	**F_1_**

ChER	77.85	78.69	78.27	73.43	57.91	64.75
ChemSpot	76.35	72.55	74.41	67.79	41.97	51.84
OSCAR4	50.88	81.34	62.60	49.90	60.73	54.79

The model trained on CHEMDNER data was proven suitable even for recognising mentions of drugs, which comprise a more specific chemical type (Table 5). When evaluated on each of the Drug-Drug Interaction (DDI) test [31,32] and Pharmacokinetics (PK) [33,34] corpora, more than satisfactory F_1 _scores (≈83%) were obtained. ChemSpot's F_1 _score on the DDI test corpus trails behind by only two percentage points, but is significantly lower than ChER's on the PK corpus with a margin of almost 10 percentage points. Applying the same model to the NaCTeM Metabolites corpus, however, did not yield results as satisfactory as those on the drug corpora, with the highest F_1 _score being 73.07% (Table 6). This, nevertheless, still indicates a significant advantage over ChemSpot, whose F_1 _score is 8 percentage points behind.

**Table 5:** Applicability of ChER with the CHEMDNER model to drug corpora.

	DDI test			PK		
	**P**	**R**	**F_1_**	**P**	**R**	**F_1_**

ChER	75.88	92.05	83.18	79.83	88.34	83.87
ChemSpot	73.09	89.49	80.46	65.29	86.07	74.25
OSCAR4	60.20	85.51	70.66	42.65	81.71	56.04

**Table 6:** Applicability of ChER with the CHEMDNER model to the NaCTeM Metabolites corpus.

	NaCTeM Metabolites		
	**P**	**R**	**F_1_**

ChER	65.08	83.29	73.07
ChemSpot	58.02	73.99	65.04
OSCAR4	35.37	84.18	49.81

Whilst the model obtained balanced precision and recall on the chemical corpus SCAI-100 (P = 77.85% vs. R = 78.69%), the suboptimal precision values on the DDI (P = 75.88% vs. R = 92.05%), PK (P = 79.83% vs. R = 88.34%) and Metabolites (P = 65.08% vs. R = 83.29%) corpora are noticeable. This drop in precision is to be expected, and can be explained by the differences between the annotation scopes of the training data, CHEMDNER, and of each of the latter three evaluation corpora. Both of the DDI and PK corpora contain only drug name annotations, whilst only metabolite mentions were annotated in the Metabolites corpus. Whereas the model was trained to recognise all chemical mentions, each of the DDI, PK and Metabolites corpora considers only a subset of them as correct, leading to an increase in the number of false positives.

### Configurable chemical entity recognition workflows in Argo

In order to facilitate the reproduction of results and further experimentation, we have made the presented named entity recognition methods available in our publicly accessible, Web-based, text mining workbench Argo [35]. The workbench aims to bring text mining to non-technical audiences by providing a graphical user interface for building and running custom text-processing applications. Applications are built in Argo visually as block diagrams forming processing pipelines, or more generally, workflows. Individual blocks in a diagram correspond to elementary processing components that are selected by users from the available, ever-growing library of analytics. The components in the library range from simple data (de)serialisers to syntactic and semantic analytics to user-interactive components.

The proposed recogniser is available as a single component and exposes multiple configurations to choose from. Users may select one of chemical, drug or metabolite, as the model that will be used for the recognition. Additional options include the disabling of post-processing steps discussed in the next section.

**Figure 1:**
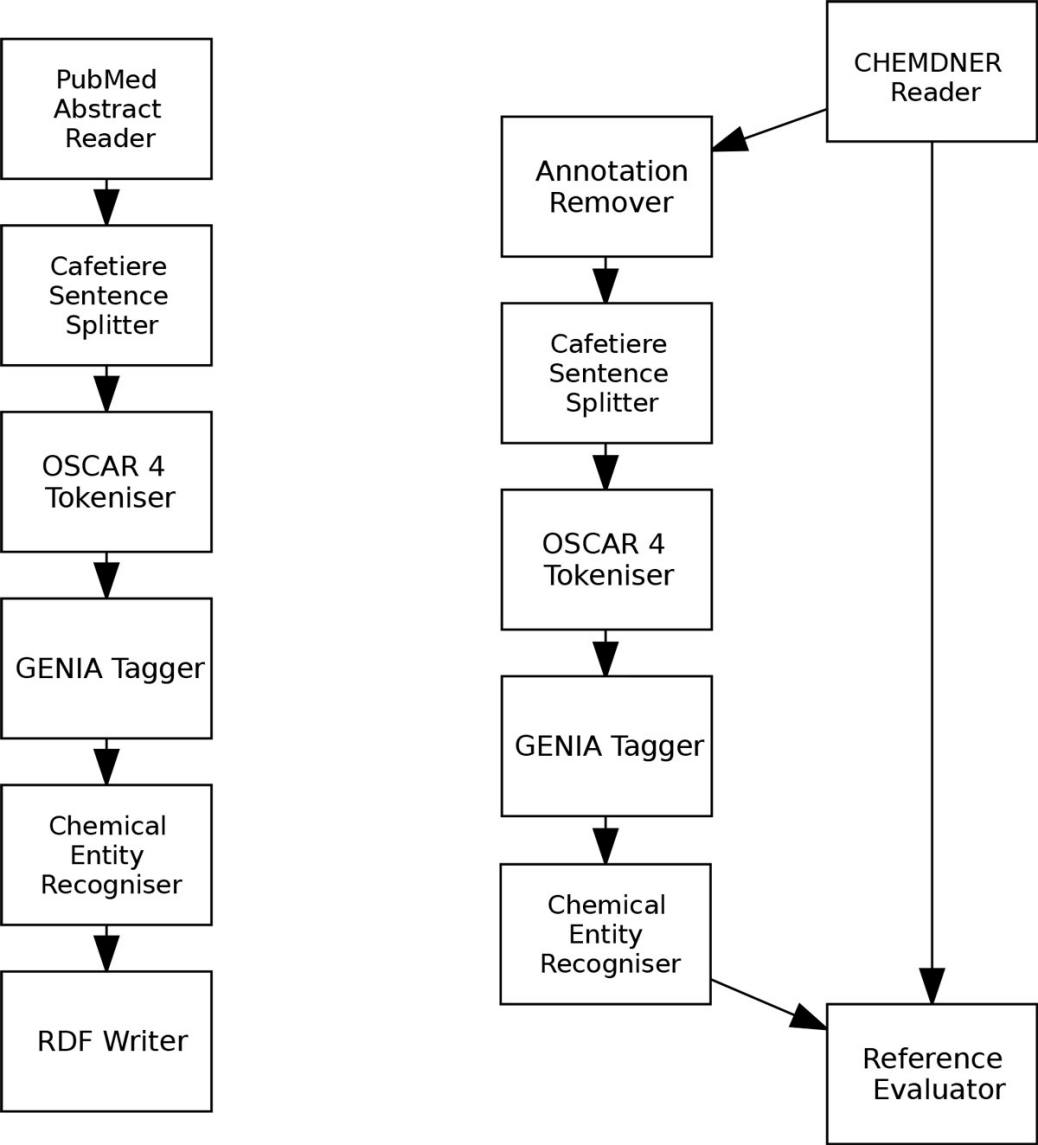
**The chemical entity recogniser in Argo**. The proposed chemical entity recogniser is available as a processing component in the Web-based, text mining workbench Argo. The component is shown here as part of two individual workflows. The left-hand-side workflow produces an RDF file containing annotated chemicals in user-specified PubMed abstracts. The right-hand-side workflow reports effectiveness metrics for the CHEMDNER corpus.

Figure 1 shows how the chemical entity recogniser component can be used in Argo workflows. Both workflows shown in the figure contain components that proved to yield the best performance on the CHEMDNER corpus. The left-hand-side workflow is set up to process PubMed articles (supplied by specifying abstract identifiers in the reader's configuration) and save the result of processing (recognised chemical names) in an RDF file. The right-hand-side workflow is a sample set up for experimenting with components available in Argo. The ultimate component in this workflow, Reference Evaluator, reports evaluation metrics based on two inputs: the reference input, which in this workflow comes directly from the CHEMDNER corpus reader and contains golden annotations, and the other branch in the workflow that attempts to reproduce the annotations in the input corpus. Users may experiment with this workflow by replacing the components (specifically the preprocessing components) with other, similar-purpose analytics available in Argo.

## Experiments

The following is a detailed description of our proposed methods and the experiments carried out to facilitate the identification of the most optimal chemical NER solutions.

### Selection of pre-processing analytics

Coming from a specialised domain, chemical literature exhibits unique properties, e.g., unusually long names, which are not typically encountered in documents from other subject domains. Whilst pre-processing steps to text mining have not been given much attention, we argue that the selection of suitable analytics for preprocessing chemical documents brings about a significant impact on NER performance, inspired by the findings of a prior exploratory work [36]. This is especially relevant in our case where features employed in training our CRF models were extracted at the basic level of tokens. In this work, we focus on the two pre-processing tasks of sentence boundary detection and tokenisation. For each of these, specialised and non-specialised implementations were explored.

#### Sentence splitters

In segmenting documents into sentences, two heuristics-based tools, i.e., the LingPipe Indo-European sentence model [37] and NaCTeM's Cafetiere sentence splitter [38], were individually employed in our experiments. Whilst the former was tuned for documents written in general language, the latter was designed specifically for scientific text, having been enriched with specialised rules that, for instance, account for the possibility of sentences beginning with lower-case characters, as with protein names, e.g., *p53*.

#### Tokenisers

For the decomposition of each sentence into tokens, we explored each of the tokenisers built into the GENIA tagger [39] and the OSCAR4 NER tool [40]. The former employs a statistical model trained on biomedical documents, whilst the latter applies segmentation rules specifically tuned for chemical texts. The OSCAR 4 tokeniser, for example, is capable of keeping intact long chemical names (e.g., *4,9-Diazadodecane-1,12-diamine*).

### Model training using a chemical knowledge-rich feature set

In building a model, we employed NERsuite, a combination of tools that include a CRF implementation [41] and utilities for embedding custom dictionary features. The following sections describe the features we used with this tool. They include basic, weakly chemical-indicative features and chemical-specific features.

#### Weakly chemical-indicative features

By default, NERsuite extracts the character and word *n*-gram features presented in Table 7. To exemplify, we provide the tokenised sentence in Table 8 as sample input, with *GSK214a *as the active token, i.e., the token currently under consideration. We note that the extraction of the word *n*-grams was done within a distance of two from the active token. Aside from these features, a token's symbol-level composition is also captured by means of the orthographic features listed in Table 9. We have augmented this set with the following:

**Table 7:** Character and word *n*-gram features extracted by NERsuite by default.

Feature	Brief description	Sample features (bigrams)
Character *n*-grams	the set of all possible combinations of a token's consecutive characters, taken n at a time (n = 2, 3, 4)	{*GS*}, {*SK*}, {*K2*}, {*21*}, {*14*}, {*4a*}

Token *n*-grams	unigrams and bigrams of surface forms; unigrams and bigrams of normalised surface forms where numbers numbers are replaced with '0's, the consecutive instances of which are compressed	{*It, attenuated*}, {*attenuated, GSK214a*}; {*Aa, aaaaaaaaaa*}, {*aaaaaaaaaa, AAA000a*}

Lemma *n*-grams	unigrams and bigrams of lemmatised surface forms	{*It, attenuate*}, {*attenuate, GSK214a*}

POS tag *n*-grams	unigrams and bigrams of part-of-speech (POS) tags	{*PRP, VBD*}, {*VBD, NN*},

Lemma & POS tag *n*-grams	unigrams and bigrams of lemmatised forms combined with POS tags	{*It:PRP, attenuate:VBD*}, {*attenuate:VBD, GSK214a:NN*}

Chunk information	chunk tag of current token; surface form of the enclosing chunk's	{*B-NP*}; {*gestation*}

**Table 8:** Example of a sentence tokenised and labelled with part-of-speech and chunk tags.

Surface form	Lemma	Part-of-speech tag	Chunk tag
*It*	It	PRP	B-NP
*attenuated*	attenuate	VBD	B-VP
***GSK214a***	GSK214a	NN	B-NP
*-induced*	-induced	JJ	I-NP
*gestation*	gestation	NN	I-NP
*in*	in	IN	B-PP
*rats*	rat	NN	B-NP
.	.	.	O

**Table 9:** Orthographic features extracted by NERsuite by default.

Feature	Example
Initial letter is in uppercase	*Boc-L-leucine*
Contains only digits	*206553 *
Contains digits	*5-HTP*
Contains only alphanumeric characters	*HClO4*
Contains only uppercase letters and digits	*AFB1 *
Contains only uppercase letters	*NO*
Does not contain any lowercase letters	*SKF81297*
Contains non-initial uppercase letters	*PbS*
Contains two consecutive uppercase letters	*PAHs*
Has a Greek letter name as a substring	*alpha-ketoacid*
Contains a comma	*3,14-dibromo*
Contains a full stop	*In(0.2)Ga(0.8)As*
Contains a hyphen	*HP-β-CD*
Contains a forward slash	*(E/Z)-Goniothalamin*
Contains an opening square bracket	*[(14)C]pazopanib*
Contains a closing square bracket	*pyrido[3,2-d]pyrimidines*
Contains an opening parenthesis	*I3 (-)*
Contains a closing parenthesis	*Fe(C10 H15)2*
Contains a semi-colon	*R = Me, Et; X = O, S;*
Contains a percentage symbol	*85%*
Contains an apostrophe	*5-methyl-2'-deoxycytidine*

Occurrence of Greek characters. This feature reflects an observation that several chemical names contain Greek characters, e.g., *(S)-α,ε-diaminohexonoic acid*.

Word shape. The active token is transformed to a representation in which numericals are converted to the '0' characters, uppercase letters to the 'A' characters, lowercase letters to the 'a' characters and everything else to the '_' characters. Full and brief word shape variants were extracted for each token. In the former, each character in the resulting representation is retained, whereas consecutive similar character types are collapsed into one in the latter. For example, the name *10-amino-20(S)-camptothecin *would have *00_aaaaa_00_A_ _aaaaaaaaaaaa *and *0_a_0_A_a *as its full and brief word shapes, respectively.

#### Chemical dictionary matches

Recognising that the occurrence of a token in an expert-curated dictionary indicates a high likelihood of it being a chemical name constituent, we utilised matches between token surface forms in text and entries in well-known chemical resources. Five dictionaries were compiled based on the chemical names and synonyms available in the Chemical Entities of Biological Interest (ChEBI) database [26], DrugBank [42], the Comparative Toxicogenomics Database (CTD) [43], PubChem Compound [44] and the Joint Chemical Dictionary (Jochem) [17]. The dictionary tools available in the NERsuite package were employed in the compilation and subsequent application of these dictionaries. We configured the compiler utility to generate a compiled dictionary whose entries were normalised by the conversion of alphabetic characters to their lower-case equivalents, numericals to the '0' characters and special characters/punctuation to the '_' characters. In the matching phase, the dictionary tagging tool performs the same conversion step on input text and then captures longest possible matches between the normalised token sequences and dictionary entries. The dictionary tagging results, exemplified in Table 10, were encoded in the begin-inside-outside (BIO) format. For an active token, unigrams and bigrams formed based on the BIO labels (within a distance of 2), as well as their combination with the corresponding surface forms, were generated as features. The token *starch*, for instance, would have the following as some of its CTD dictionary features: {*on:O*, *hydroxyethyl:B*}, {*hydroxyethyl:B*, *starch:I*} as surface form and dictionary label bigrams, and {*O*, *B*}, {*B*, *I*} as dictionary label bigrams.

**Table 10:** Example of a token sequence tagged with matches against chemical dictionaries.

Token	Normal form	ChEBI	DrugBank	CTD	PubChem	Jochem
*For*	for	O	O	O	O	O
*the*	the	O	O	O	O	O
*preparation*	preparation	O	O	O	O	O
*of*	of	O	O	O	O	O
*hydrogel*	hydrogel	O	O	B	O	B
*microspheres*	microsphere	O	O	O	O	O
*based*	base	O	O	O	O	O
*on*	on	O	O	O	O	O
*hydroxyethyl*	hydroxyethyl	O	O	B	O	B
***starch***	starch	B	O	I	O	I
*-*	_	B	O	O	O	O
*hydroxyethyl*	hydroxyethyl	I	O	B	O	B
*methacrylate*	methacrylate	I	O	I	B	I
*(*	_	O	O	O	O	O
*HES-HEMA*	hes_hema	O	O	O	O	O
*)*	_	O	O	O	O	O

#### Chemical affix matches

Many of the chemical names, especially nomenclature-based ones, contain chemical affixes (i.e., prefixes and suffixes). We attempt to capture this property by matching tokens in text against lists of commonly used chemical prefixes and suffixes whose lengths range from two to four. Shown in Table 11 is a sequence of tokens matched against our compiled affix lists, which are provided in an additional file (see Additional file 1). The feature set is augmented with the resulting affix matches.

**Table 11:** Example of a token sequence tagged with matches against our affix lists.

	Prefixes			Suffixes		
**Token**	**size 2**	**size 3**	**size 4**	**size 2**	**size 3**	**size 4**

*Incubation*	O	O	O	O	O	O
*with*	O	O	O	O	O	O
*diisopropyl*	di	O	O	yl	O	O
*fluorophosphate*	O	O	fluo	O	ate	O
*and*	O	O	O	O	O	O
*bis-(4-nitrophenyl)*	O	O	O	O	O	O
*phosphate*	O	O	O	O	ate	O

#### Number of chemical basic segments

Nomenclature-based chemical expressions, e.g., systematic and semi-systematic names, are formed from combinations of chemical segments. These segments are documented in the American Chemical Society's Registry File Basic Name Segment Dictionary, which contains a total of 3,307 entries as well as a description of the procedure for decomposing a name into its basic segments [45]. Following this algorithm, we process the surface form of each token to determine the number of constituent chemical basic segments. Table 12 lists the basic chemical segments found within the given expressions. We note that the number of basic segments also includes fragments which remain unmatched against the segment dictionary. For instance, only the fragments *methyl*, *ergo *and *novi *in the name *methylergonovine *can be found by the procedure; however, the remaining fragment *ne *was also counted as a basic segment.

**Table 12:** Examples of chemical names with corresponding basic segments.

Token	Basic segments	No. of basic segments
*10-acetoxyactinidine*	10, acet, oxy, actin, idine	5

*methylergonovine*	methyl, ergo, novi, ne	4

*interleukin-2*	interleukin, 2	2

#### Chemical symbol matches

In order to account for chemical element symbols, which are not always covered by our five chosen dictionaries, we matched tokens in text against a list of symbols which occur in the periodic table of elements. This list has been provided in an additional file (see Additional file 2).

### Heuristics-based post-processing

Two CRF models were initially learned from the CHEMDNER training corpus: one with only the default features and another with our engineered ones. For each input sentence, each of the models automatically generates a label sequence in BIO format, together with the confidence values with which the labels were assigned. Upon individually applying the models on the CHEMDNER development set, we obtained the results presented in Table 13. Whilst the performance boost brought about by our customised features was encouraging, the suboptimal recall prompted us to introduce post-processing steps for reducing the number of false negatives.

**Table 13:** Performance of models learned from the CHEMDNER training set when evaluated on the development set.

	Macro			Micro		
	**P**	**R**	**F_1_**	**P**	**R**	**F_1_**

Default features	86.66	79.01	80.89	88.55	76.82	82.27
Enriched features	88.26	81.11	82.86	89.87	78.99	84.07

Margin	+1.6	+2.1	+1.97	+1.32	+2.17	+1.8

By inspecting the distribution of false negatives according to chemical mention types (provided in Table 14), we identified the most prevalent problematic cases which we addressed with two rule-based post-processing steps.

**Table 14:** Distribution (according to chemical subtype) of the instances incorrectly rejected by the model trained with enriched features.

Subtype	Frequency	Percentage
Abbreviation	1,882	30.32%
Formula	1,291	20.80%
Family	979	15.77%
Trivial	926	14.92%
Systematic	693	11.16%
Identifier	293	4.72%
Multiple	118	1.90%
No class	25	0.40%

#### Abbreviation recognition

To alleviate the problem of missed abbreviations which account for about 30% of the false negatives, we introduced an abbreviation recognition step which performs the following checks given the last token *t_i _*of a named entity *e *recognised by the CRF model:

• *t*_*i*+1 _is the opening parenthesis '(',

• *t*_*i*+3 _is the closing parenthesis ')', and

• *t*_*i*+2 _was recognised as a non-chemical token by the CRF model.

Token *t*_*i*+2 _becomes a candidate abbreviation for *e *if all three conditions hold true. As a verification step, a procedure [46] for checking the sequential occurrence of each character in *t*_*i*+2 _within the entity *e *is performed. Upon successful verification, all instances of token *t*_*i*+2 _within the document are relabelled as chemical tokens. In this manner, for instance, the chemical abbreviation *STMP *missed by the CRF model will be captured from the phrase, "*... was phosphorylated with sodium trimetaphosphate (STMP) at ambient temperature..*." assuming that *sodium trimetaphosphate *was recognised by the model as a chemical entity.

#### Chemical composition-based token relabelling

About 42% of the false negatives correspond to missed family, trivial and systematic names, all of which typically contain chemical segments. In attempting to increase recall for these mention types, we developed a procedure that analyses tokens which were labelled by the CRF model as non-chemical with confidence values lower than a chosen threshold *t*_1_. These tokens are of interest as the relatively low confidence values attached to them indicate their likelihood of being chemical name constituents. This likelihood was further verified by the computation of a token's chemical segment composition, given by the ratio of the number of characters comprising segments matched against the chemical basic segment dictionary to the total number of characters in the token. Sample tokens and the ratios calculated for them are provided in Table 15. The procedure relabels a token of interest as chemical if its chemical segment composition is greater than a chosen threshold *t*_2_.

**Table 15:** Sample tokens and their chemical segment composition.

Token initially recognised as non-chemical	Chemical basic segments	Ratio
*polycalcium*	poly, calcium	1.0

*2-methoxyestradiol*	meth, oxy, estra, di, ol	0.89

*palytoxin*	toxin	0.56

Different combinations of the thresholds *t*_1 _and *t*_2 _were investigated to establish the most optimal values. After a few exploratory runs, we decided to restrict our search space to the range [0.91, 0.99] for *t*_1 _and to [0.5, 0.9] for *t*_2_. These were exhaustively probed in increments of 0.01 and 0.1 for *t*_1 _and *t*_2_, respectively, by means of evaluation on the CHEMDNER development data set. Results showed that recall is optimal with *t*_1 _= 0.96 and *t*_2 _= 0.5, and that optimal precision and F_1 _scores are obtained with *t*_1 _= 0.93 and *t*_2 _= 0.9. In the rest of the experiments presented in this paper, we used the latter threshold settings.

### Evaluation

With the proposed extensions presented above, chemical NER can be optimised according to the following five dimensions:

1 Pre-processing: Sentence splitting (LingPipe Indo-European model or Cafetiere)

2 Pre-processing: Tokenisation (GENIA or OSCAR4)

3 Model training: Knowledge-rich features (include or exclude)

4 Post-processing: Abbreviation recognition (enable or disable)

5 Post-processing: Chemical composition-based token relabelling (enable or disable)

Out of the possible combinations from these five dimensions, we selected 20 for each of our experiments, enabling abbreviation recognition and chemical composition-based token relabelling only when knowledge-rich features were employed in model training. This comprehensive evaluation was carried out with the utilisation of the CHEMDNER, SCAI and SciBorg corpora, as well as the following document collections:

Patents [30]. This corpus is the outcome of the collaborative effort of curators from the European Patent Office and the ChEBI project who annotated all mentions of chemical entities in 40 patent application documents [29].

Drug-Drug Interaction (DDI) [32]. Consisting of 233 MEDLINE abstracts and 792 textual descriptions from the DrugBank database, this corpus contains annotated drug mentions pertaining to generic names, brands, groups (e.g., *antibiotic*) and non-human applications (e.g., *pesticide*) [31]. Released as a resource for the SemEval 2013 DDI Extraction task [47], the corpus is divided into subsets for training and testing.

Pharmacokinetics (PK) [34]. Also containing drug name annotations, this corpus is comprised of a selection of 541 MEDLINE abstracts on the topics of clinical pharmacokinetics and phamacogenetics as well as *in vitro *and *in vivo *drug-drug interactions [33].

NaCTeM Metabolites [28]. This document collection contains 296 MEDLINE abstracts with annotations for metabolite and enzyme names [23]. For our evaluation, only metabolite name annotations were taken into consideration.

Table 16 summarises the results of the best performing ChER combination in each of the experiments we conducted. For the purpose of comparison, we have also provided results obtained by our baseline, i.e., the variant of the named entity recogniser that employs non-specialised pre-processing analytics (i.e., the LingPipe Indo-European sentence model and the GENIA tokeniser) and none of the knowledge-rich features and post-processing heuristics. It can be observed that in majority of the nine sets of experiments in this table, the optimal combination for ChER incorporates the use of specialised pre-processing tools, feature set enrichment and abbreviation recognition. The lack of a unique combination yielding optimal results across all evaluation data sets can be explained by the differences of the corpora in terms of the guidelines which were adhered to during their annotation. Enabling chemical composition-based token relabelling brought about improved F_1 _scores on the SciBorg, Patents, Metabolites and DDI corpora (owing to increased recall), but resulted in lower values of F_1 _on the CHEMDNER, SCAI and PK corpora (due to decreased precision). This post-processing step, for example, captured mentions of anions which were considered as chemical names in the SciBorg and Metabolites corpora (e.g., *silicate*, *glutamate*, *succinate*) but were counted as false positives by the SCAI corpus. Similarly, some chemical umbrella terms, such as *esters *and *nucleotides*, captured by this step were treated as true positives under evaluation against the Patents corpus, but stand for false positives in the CHEMDNER, SCAI and PK corpora. Another source of discrepancy are chemical named entities which have ambiguous meanings, that this rule-based step is oblivious to. *Iron *as a metallic element, for example, was not annotated in CHEMDNER and SCAI, but is considered a drug (i.e., a vitamin) in the DDI corpus. Meanwhile, abbreviation recognition boosted ChER's performance on all corpora except for the DDI corpus, where no impact was observed due to it not having been annotated with abbreviation information.

**Table 16:** Summary of ChER's performance under the CHEMDNER track setting (set 1), under similar experimental settings as state-of-the-art methods (sets 2-4), and when applied to various corpora (sets 5-9).

	Data		Pre-processing		**Cust**.	Post-processing		Micro-averages		
	Training	Test	Splitter	Tokeniser	**Feats**.	**Abbr**.	**Comp**.	P	R	F_1_
1	CHEMDNER	CHEMDNER	LingPipe	GENIA	✗	✗	✗	88.87	70.95	78.91
	training & dev.	test	Cafetiere	OSCAR4	✓	✓	✓	92.76	81.30	86.65

2	SciBorg (CM):3-fold CV		LingPipe	GENIA	✗	✗	✗	80.44	55.16	65.45
			Cafetiere	OSCAR4	✓	✓	✓	85.96	74.22	79.66

3	SCAI-IUPAC	SCAI-100	LingPipe	GENIA	✗	✗	✗	84.78	66.87	74.77
	training	(IUPAC)	Cafetiere	GENIA	✓	✓	✓	86.70	67.50	75.90
	
4	NaCTeM Metabolites:10-fold CV		LingPipe	GENIA	✗	✗	✗	81.72	64.49	72.09
			Cafetiere	OSCAR4	✓	✓	✓	81.42	79.66	80.53

5	CHEMDNER	SCAI-100	LingPipe	GENIA	✗	✗	✗	72.56	66.00	69.13
	training & dev.	(All)	Cafetiere	OSCAR4	✓	✓	✓	77.85	78.69	78.27

6	CHEMDNER	Patents	LingPipe	GENIA	✗	✗	✗	72.66	52.97	61.27
	training & dev.		Cafetiere	OSCAR4	✓	✓	✓	73.43	57.91	64.75

7	CHEMDNER	DDI	LingPipe	GENIA	✗	✗	✗	76.52	75.00	75.75
	training & dev.	test	Cafetiere	OSCAR4	✓	•	✓	75.88	92.05	83.18

8	CHEMDNER	PK	LingPipe	GENIA	✗	✗	✗	79.29	84.66	81.89
	training & dev.		Cafetiere	GENIA	✓	✓	✓	79.83	88.34	83.87

9	CHEMDNER	NaCTeM	LingPipe	GENIA	✗	✗	✗	63.57	71.63	67.36
	training & dev.	Metabolites	Cafetiere	OSCAR4	✓	✓	✓	65.08	83.29	73.07

The first row in each set corresponds to the baseline. Key: Cust. Feats. = Custom Features, Abbr. = Abbreviation recognition, Comp. = Chemical composition-based token relabelling; ✓ = enabled, ✗ = disabled, • = enabling or disabling makes no difference in performance.

The results of all 20 combinations, in each of the nine experimental set-ups described in Table 16, are provided in Additional file 3. The impact on performance of individually selecting a particular pre-processing analytic or enabling a specific post-processing heuristic can be easily observed from this file. For example, on the CHEMDNER test data, the ChER variant that employs Cafetiere Sentence Splitter, OSCAR4 Tokeniser, knowledge-rich features and abbreviation recognition for the CEM task obtains an F_1 _score of 86.65%. Replacing OSCAR4 Tokeniser with GENIA Tokeniser, however, leads to a 6-percentage point drop in F_1 _score (80.1%).

## Conclusions

The exhaustive evaluation of our proposed tool ChER shows that in majority of cases the most optimal variant incorporates specialised pre-processing analytics (specifically, the Cafetiere sentence splitter and OSCAR4 tokeniser), knowledge-rich machine-learning features and a post-processing step for abbreviation recognition. In each experiment that we performed, comparison of the optimal combination with the baseline (i.e., the variant of the NER without any of our proposed additions) indicates noticeably better performance of the former over the latter. When compared to state-of-the-art methods, our solutions obtain competitive, if not superior, performance.

ChER with a statistical model learned from the training and development sets of the CHEMDNER corpus proved to achieve a satisfactory performance on a variety of corpora, regardless of document type and chemical subdomain, consistently outperforming the state of the art.

As our solutions are all accessible and usable via the Argo text mining platform, interested parties can replicate our results, if not introduce further improvements to our solution by exploring other analytics. Moreover, owing to the interoperable nature of Argo, our chemical entity recogniser, ChER, does not impose any restrictions in terms of input and output formats. It can be easily integrated as a semantic analytic in other text mining tasks such as document indexing and entity relation extraction.

## Methods

### Sequence labelling

In addressing the problem of named entity recognition, we employed a sequence labelling approach which involves the automatic assignment of labels to a given sequence of items, i.e., the ordered tokens in a sentence. The set of possible labels was defined by our chosen encoding scheme, the begin-inside-outside (BIO) representation. This scheme uses the labels 'B' and 'I' to indicate the beginning and continuing tokens of a chemical name, respectively, and 'O' to mark tokens which are not part of any chemical name. To transform the documents into this representation, the following pre-processing pipeline was applied on raw input text:

#### Sentence splitting

Text contained in each document was segmented by means of a sentence splitter. As described previously, the LingPipe Indo-European sentence model and Cafetiere sentence splitter were individually applied in this work.

#### Tokenisation

In segmenting each sentence into tokens, we utilised each of the GENIA and OSCAR4 tokenisers in our experiments.

Part-of-speech and chunk tagging. Each resulting token is automatically lemmatised and assigned tags which correspond to its part-of-speech (POS) and enclosing chunk. This information was supplied by the GENIA Tagger [39] which employs maximum entropy models in analysing both general and biomedical-domain documents. Shown in Table 8 are the lemmata, POS and chunk tags assigned to the tokens of the given sentence.

Our sequence labelling approach was realised as an application of the machine learning-based conditional random fields algorithm (CRFs). Given an item sequence, a CRF model predicts the most probable label sequence based on functions capturing characteristics of the current token and its context. These functions, typically referred to as features (discussed in detail in the Experiments section), are employed in both training and prediction phases. We built our named entity recognisers on top of the NERsuite package [48], an implementation of CRFs with a built-in extractor of features typically used in biomedical NER.

### Evaluation metrics

We reported the effectiveness of our methods with the commonly used information retrieval metrics, namely, precision (P), recall (R) and F_1 _score defined as follows:

$$P=\frac{TP}{TP+FP},\;R=\frac{TP}{TP+FN},\;F_{1}=\frac{2*P*R}{P+R},$$

where *TP*, *FP *and *FN *are the numbers of, respectively, true positive, false positive and false negative recognitions. Intuitively, precision is the fraction of recognised entities that are correct, recall is the fraction of manually annotated entities that were recognised, and F_1 _is a balanced harmonic mean between the two. F_1 _represents a more conservative metric than the arithmetic average.

We note that all of the results reported in this paper, including those of the other chemical NER tools, were obtained using the evaluation tool provided by the BioCreative organisers [49]. The tool calculates the macro- and micro-averaged values of the aforementioned metrics.

## Competing interests

The authors declare that they have no competing interests.

## Authors' contributions

RB carried out the development and evaluation of the proposed methods for chemical NER optimisation. RR developed the various components for making the results of this work available and usable in Argo. Both RR and RB formulated the study. SA provided guidance in the evaluation of the work and coordinated the effort. All authors read and approved the final manuscript.

## Supplementary Material

Additional file 1 **List of chemical affixes**. A listing of the most common chemical prefixes and suffixes. Click here for file

Additional file 2 **List of chemical element symbols**. A listing of the chemical element symbols. Click here for file

Additional file 3 **Chemical Entity Recogniser (ChER) Experiments**. Tables containing the results of nine experiments, each comparing 20 combinations of the proposed methods. Click here for file

## Declaration

Funding for this article's publication was granted by the University of Manchester's Wellcome Trust award.

This article has been published as part of *Journal of Cheminformatics *Volume 7 Supplement 1, 2015: Text mining for chemistry and the CHEMDNER track. The full contents of the supplement are available online at http://www.jcheminf.com/supplements/7/S1.

## References

[B1] Davis AP Wiegers TC Johnson RJ Lay JM Lennon-Hopkins K Saraceni-Richards C Sciaky D Murphy CG Mattingly CJ Text mining effectively scores and ranks the literature for improving chemical-gene-disease curation at the comparative toxicogenomics database PLoS ONE 2013 8 4 58201 10.1371/journal.pone.0058201 PMC3629079 23613709

[B2] Kolářik C Hofmann-Apitius M Banville DL Linking Chemical and Biological Information with Natural Language Processing Chemical Information Mining 2009 Chap 7 123 150

[B3] Segura-Bedmar I Martínez P de Pablo-Sánchez C Extracting drug-drug interactions from biomedical texts BMC Bioinformatics 2010 11 S-5 920053276

[B4] Deftereos SN Andronis C Friedla EJ Persidis A Persidis A Drug repurposing and adverse event prediction using high-throughput literature analysis. Wiley interdisciplinary reviews Systems biology and medicine 2011 3 3 323 34 21416632 10.1002/wsbm.147

[B5] Li C Liakata M Rebholz-Schuhmann D Biological network extraction from scientific literature: state of the art and challenges Briefings in Bioinformatics 2013 23434632 10.1093/bib/bbt006

[B6] Banville DL Mining chemical structural information from the drug literature Drug Discovery Today 2006 11 1 35 42 16478689 10.1016/S1359-6446(05)03682-2

[B7] Vazquez M Krallinger M Leitner F Valencia A Text mining for drugs and chemical compounds: Methods, tools and applications Molecular Informatics 2011 30 6-7 506 519 10.1002/minf.201100005 27467152

[B8] Gurulingappa H Mudi A Toldo L Hofmann-Apitius M Bhate J Challenges in mining the literature for chemical information RSC Adv 2013 16194 16211

[B9] Grego T Pesquita C Bastos HP Couto FM Chemical Entity Recognition and Resolution to ChEBI ISRN Bioinformatics 2012 2012 9 10.5402/2012/619427 PMC4393067 25937941

[B10] Corbett P Batchelor C Teufel S Annotation of chemical named entities Proceedings of the Workshop on BioNLP 2007: Biological, Translational, and Clinical Language Processing. BioNLP '07 2007 Association for Computational Linguistics, Stroudsburg, PA, USA 57 64

[B11] Chemistry Using Text Annotations http://nactem.ac.uk/cheta Accessed: October 2013

[B12] Rebholz-Schuhmann D Yepes J Jose A Van Mulligen EM Kang N Kors J Milward D Corbett P Buyko E Beisswanger E Hahn U CALBC silver standard corpus Journal of Bioinformatics and Computational Biology 2010 8 1 163 79 10.1142/S0219720010004562 20183881

[B13] Kolářik C Klinger R Friedrich CM Hofmann-Apitius M Fluck J Chemical names: Terminological resources and corpora annotation Proceedings of the Workshop on Building and Evaluating Resources for Biomedical Text Mining. LREC 2008 51 58

[B14] Fraunhofer SCAI Corpora for Chemical Entity Recognition http://www.scai.fraunhofer.de/chem-corpora.html Accessed: October 2013

[B15] Corbett P Copestake A Cascaded classifiers for confidence-based chemical named entity recognition BMC Bioinformatics 2008 9 Suppl 11 4 10.1186/1471-2105-9-S11-S4 19025690 PMC2586753

[B16] Rocktäschel T Weidlich M Leser U ChemSpot: a hybrid system for chemical named entity recognition Bioinformatics 2012 28 12 1633 1640 10.1093/bioinformatics/bts183 22500000

[B17] Hettne KM Stierum RH Schuemie MJ Hendriksen PJM Schijvenaars BJA van Mulligen EM Kleinjans J Kors JA A dictionary to identify small molecules and drugs in free text Bioinformatics 2009 25 22 2983 2991 10.1093/bioinformatics/btp535 19759196

[B18] Lafferty JD McCallum A Pereira FCN Conditional Random Fields: Probabilistic Models for Segmenting and Labeling Sequence Data Proceedings of the Eighteenth International Conference on Machine Learning. ICML '01 2001 Morgan Kaufmann Publishers Inc., San Francisco, CA, USA 282 289

[B19] Krallinger M Leitner F Rabal O Vazquez M Oyarzabal J Valencia A CHEMDNER: The drugs and chemical names extraction challenge J Cheminform 2015 7 Suppl 1 S1 10.1186/1758-2946-7-S1-S1 PMC4331685 25810766

[B20] Krallinger M Rabal O Leitner F Vazquez M Salgado D Lu Z Leaman R Lu Y Ji D Lowe DM Sayle RA Batista-Navarro RT Rak R Huber T Rocktaschel T Matos S Campos D Tang B Xu H Munkhdalai T Ryu KH Ramanan SV Nathan S Zitnik S Bajec M Weber L Irmer M Akhondi SA Kors JA Xu S An X Sikdar UK Ekbal A Yoshioka M Dieb TM Choi M Verspoor K Khabsa M Giles CL Liu H Ravikumar KE Lamurias A Couto FM Dai H Tsai RT Ata C Can T Usie A Alves R Segura-Bedmar I Martinez P Oryzabal J Valencia A The CHEMDNER corpus of chemicals and drugs and its annotation principles J Cheminform 2015 7 Suppl 1 S2 10.1186/1758-2946-7-S1-S2 PMC4331692 25810773

[B21] Rak R Batista-Navarro RT Carter J Rowley A Ananiadou S Processing biological literature with customizable web services supporting interoperable formats Database 2014 2014 064 25006225 10.1093/database/bau064 PMC4086403

[B22] Batista-Navarro RTB Rak R Ananiadou S Chemistry-specific features and heuristics for developing a CRF-based chemical named entity recogniser Proceedings of the Fourth BioCreative Challenge Evaluation Workshop 2013 2 55 59

[B23] Nobata C Dobson PD Iqbal SA Mendes P Tsujii J Kell DB Ananiadou S Mining metabolites: extracting the yeast metabolome from the literature Metabolomics 2011 7 1 94 101 10.1007/s11306-010-0251-6 21687783 PMC3111869

[B24] OSCAR4 https://bitbucket.org/wwmm/oscar4/wiki/Home Accessed: October 2013

[B25] ChemSpot https://github.com/rockt/ChemSpot Accessed: October 2013

[B26] Hastings J de Matos P Dekker A Ennis M Harsha B Kale N Muthukrishnan V Owen G Turner S Williams M Steinbeck C The ChEBI reference database and ontology for biologically relevant chemistry: enhancements for 2013 Nucleic Acids Research 2012 23180789 10.1093/nar/gks1146 PMC3531142

[B27] Wishart DS Knox C Guo AC Eisner R Young N Gautam B Hau DD Psychogios N Dong E Bouatra S Mandal R Sinelnikov I Xia J Jia L Cruz JA Lim E Sobsey CA Shrivastava S Huang P Liu P Fang L Peng J Fradette R Cheng D Tzur D Clements M Lewis A De Souza A Zuniga A Dawe M Xiong Y Clive D Nazyrova A Shaykhutdinov R Li L Vogel HJ Forsythe I HMDB: a knowledgebase for the human metabolome Nucleic Acids Research 2009 37 suppl 1 603 610 10.1093/nar/gkn810 PMC2686599 18953024

[B28] NaCTeM Metabolite and Enzyme Corpus http://www.nactem.ac.uk/metabolite-corpus Accessed: October 2013

[B29] Grego T Pęzik P Couto FM Rebholz-Schuhmann D Identification of chemical entities in patent documents Proceedings of the 10th International Work-Conference on Artificial Neural Networks: Part II: Distributed Computing, Artificial Intelligence, Bioinformatics, Soft Computing, and Ambient Assisted Living. IWANN '09 2009 Springer, Berlin, Heidelberg 942 949

[B30] Patents Gold Standard Annotations http://chebi.cvs.sourceforge.net/viewvc/chebi/chapati/patentsGoldStandard Accessed: October 2013

[B31] Herrero-Zazo M Segura-Bedmar I Martínez P Declerck T The ddi corpus: An annotated corpus with pharmacological substances and drug-drug interactions Journal of Biomedical Informatics 2013 46 5 914 920 10.1016/j.jbi.2013.07.011 23906817

[B32] Corpora for Drug-Drug Interaction Extraction http://labda.inf.uc3m.es/doku.php?id=en:labda_ddicorpus Accessed: October 2013

[B33] Wu H-Y Karnik S Subhadarshini A Wang Z Philips S Han X Chiang C Liu L Boustani M Rocha L Quinney S Flockhart D Li L An integrated pharmacokinetics ontology and corpus for text mining BMC Bioinformatics 2013 14 1 35 10.1186/1471-2105-14-35 23374886 PMC3571923

[B34] Pharmacokinetics Corpus http://rweb.compbio.iupui.edu/corpus Accessed: October 2013

[B35] Rak R Rowley A Black W Ananiadou S Argo: an integrative, interactive, text mining-based workbench supporting curation Database : The Journal of Biological Databases and Curation 2012 010 22434844 10.1093/database/bas010 PMC3308166

[B36] Kolluru B Hawizy L Murray-Rust P Tsujii J Ananiadou S Using workflows to explore and optimise named entity recognition for chemistry PLoS ONE 2011 6 5 20181 10.1371/journal.pone.0020181 PMC3102085 21633495

[B37] Alias-I: LingPipe 4.1.0 http://alias-i.com/lingpipe Accessed: July 2013

[B38] Cafetiere English Sentence Detector http://metashare.metanet4u.eu/repository/browse/u-compare-cafetiere-english-sentence-detector/aff1ddc0bc8911e1a404080027e73ea259aeca28412944ea97f7b2580a41caec/# Accessed: October 2013

[B39] Tsuruoka Y Tateisi Y Kim J-D Ohta T McNaught J Ananiadou S Tsujii J Developing a Robust Part-of-Speech Tagger for Biomedical Text. In: Advances in Informatics - 10th Panhellenic Conference on Informatics LNCS, Springer, Volos, Greece 2005 3746 382 392

[B40] Jessop DM Adams SE Willighagen EL Hawizy L Murray-Rust P OSCAR4: a flexible architecture for chemical text-mining Journal of Cheminformatics 2011 3 1 41 10.1186/1758-2946-3-41 21999457 PMC3205045

[B41] Okazaki N CRFsuite: a fast implementation of Conditional Random Fields (CRFs) http://www.chokkan.org/software/crfsuite Accessed: July 2013

[B42] Knox C Law V Jewison T Liu P Ly S Frolkis A Pon A Banco K Mak C Neveu V Djoumbou Y Eisner R Guo AC Wishart DS DrugBank 3.0: a comprehensive resource for 'omics' research on drugs Nucleic acids research 2011 39 Database 1035 41 21059682 10.1093/nar/gkq1126 PMC3013709

[B43] Davis AP Murphy CG Johnson R Lay JM Lennon-Hopkins K Saraceni-Richards C Sciaky D King BL Rosenstein MC Wiegers TC Mattingly CJ The Comparative Toxicogenomics Database: update 2013 Nucleic Acids Research 2012 23093600 10.1093/nar/gks994 PMC3531134

[B44] Bolton EE Wang Y Thiessen PA Bryant SH PubChem: Integrated Platform of Small Molecules and Biological Activities Annual Reports in Computational Chemistry 2008 4

[B45] American Chemical Society Registry file basic name segment dictionary. Technical report 1993

[B46] Schwartz AS Hearst MA A simple algorithm for identifying abbreviation definitions in biomedical text Pacific Symposium on Biocomputing 2003 451 462 12603049

[B47] Segura-Bedmar I Martínez P Herrero Zazo M SemEval-2013 Task 9: Extraction of Drug-Drug Interactions from Biomedical Texts (DDIExtraction 2013) Second Joint Conference on Lexical and Computational Semantics (*SEM), Volume 2: Proceedings of the Seventh International Workshop on Semantic Evaluation (SemEval 2013) 2013 Association for Computational Linguistics, Atlanta, Georgia, USA 341 350

[B48] Cho H-C Okazaki N Miwa M Tsujii J NERsuite: a named entity recognition toolkit https://github.com/nlplab/nersuite Accessed: July 2013

[B49] Leitner F BioCreative II.5 Evaluation Library http://www.biocreative.org/resources/biocreative-ii5/evaluation-library Accessed: August 2013

